# An Overview of Genetic Polymorphisms and Pancreatic Cancer Risk in Molecular Epidemiologic Studies

**DOI:** 10.2188/jea.JE20100090

**Published:** 2011-01-05

**Authors:** Yingsong Lin, Kiyoko Yagyu, Naoto Egawa, Makoto Ueno, Mitsuru Mori, Haruhisa Nakao, Hiroshi Ishii, Kozue Nakamura, Kenji Wakai, Satoyo Hosono, Akiko Tamakoshi, Shogo Kikuchi

**Affiliations:** 1 Department of Public Health, Aichi Medical University School of Medicine, Nagakute, Japan; 2 Department of Internal Medicine, Tokyo Metropolitan Komagome Hospital, Tokyo, Japan; 3 Hepatobiliary and Pancreatic Medical Oncology Division, Kanagawa Cancer Center Hospital, Kanagawa, Japan; 4 Department of Public Health, Sapporo Medical University School of Medicine, Sapporo, Japan; 5 Division of Gastroenterology, Department of Internal Medicine, Aichi Medical University School of Medicine, Nagakute, Japan; 6 Hepatobiliary and Pancreatic Section, Gastroenterological Division, Cancer Institute Hospital, Tokyo, Japan; 7 Department of Epidemiology and Preventive Medicine, Gifu University Graduate School of Medicine, Gifu, Japan; 8 Department of Preventive Medicine, Nagoya University Graduate School of Medicine, Nagoya, Japan; 9 Division of Epidemiology and Prevention, Aichi Cancer Center Research Institute, Nagoya, Japan

**Keywords:** pancreatic cancer, genetic polymorphisms, molecular epidemiology, gene–environment interaction

## Abstract

**Background:**

Although pancreatic cancer has been extensively studied, few risk factors have been identified, and no validated biomarkers or screening tools exist for early detection in asymptomatic individuals. We present a broad overview of molecular epidemiologic studies that have addressed the relationship between pancreatic cancer risk and genetic polymorphisms in several candidate genes and suggest avenues for future research.

**Methods:**

A comprehensive literature search was performed using the PubMed database.

**Results:**

Overall, individual polymorphisms did not seem to confer great susceptibility to pancreatic cancer; however, interactions of polymorphisms in carcinogen-metabolizing genes, DNA repair genes, and folate-metabolizing genes with smoking, diet, and obesity were shown in some studies. The major problem with these studies is that, due to small sample sizes, they lack sufficient statistical power to explore gene–gene or gene–environment interactions. Another important challenge is that the measurement of environmental influence needs to be improved to better define gene–environment interaction. It is noteworthy that 2 recent genome-wide association studies of pancreatic cancer have reported that variants in ABO blood type and in 3 other chromosomal regions are associated with risk for this cancer, thus providing new insight into pancreatic cancer etiology.

**Conclusions:**

As is the case in other complex diseases, common, low-risk variants in different genes may act collectively to confer susceptibility to pancreatic cancer in individuals with repeated environmental exposures, such as smoking and red meat intake. Clarification of gene–gene and gene–environmental interaction is therefore indispensable for future studies. To address these issues, a rigorously designed molecular epidemiologic study with a large sample is desirable.

## INTRODUCTION

Pancreatic cancer is a major cause of cancer mortality in developed countries.^1^ In Japan, approximately 25 000 Japanese men and women died from pancreatic cancer in 2007, making it the fifth leading cause of cancer death.^2^ Pancreatic cancer is a rapidly fatal disease, with mortality almost identical to incidence. The all-stage 5-year survival rate is less than 10%, the lowest among all cancer sites.^3^

The etiology of sporadic pancreatic cancer is not well understood. However, mounting evidence suggests that pancreatic carcinogenesis involves a complex interaction between genetic mutations, epigenetic alterations, and environmental risk factors.^4^ Among these environmental risk factors, epidemiologic studies have identified only cigarette smoking and type II diabetes as clear risk factors for pancreatic cancer.^5^^,^^6^ An association of pancreatic cancer with dietary habits remains unclear because of wide variation in dietary habits across populations and the difficulty of accurate diet measurement.^7^

Due to the completion of human genome sequencing and rapid progress in sequencing techniques, an increasing number of studies are exploring the associations between polymorphisms in candidate genes and pancreatic cancer risk. In a search of the PubMed database using the keywords “genetic polymorphism” plus “pancreatic cancer,” we found 217 publications that had been published in the last 10 years. Furthermore, a small but growing body of research has addressed gene–environment interaction contributing to pancreatic cancer development.^8^^–^^10^ Of special importance are 2 recent genome-wide association studies (GWAS), which reported that a variant in ABO blood type and 3 other variants in chromosomal regions are associated with pancreatic cancer risk.^11^^,^^12^

An estimated 10% of pancreatic cancer cases are associated with inherited predisposition, based on familial clustering.^13^ Several germline mutations have been linked to familial pancreatic cancer.^14^ The role of germline mutations in several genes, such as *INK4A*, *BRCA2*, and *LKB1*, and their associations with pancreatic cancer risk is beyond the scope of this review. However, in this article we provide a broad overview of molecular epidemiologic studies that have investigated the relationship between genetic polymorphisms in several candidate genes and their interactions with environmental factors in conferring pancreatic cancer risk. We focus on polymorphisms in carcinogen-metabolizing genes, DNA repair genes, and folate-metabolizing genes because the functional importance of these genes has been elucidated and because most published studies have examined these genes. Furthermore, on the basis of findings from GWAS and biomarker, epidemiologic, and experimental studies, we identify additional genetic polymorphisms that require analysis due to their potentially important role in the etiology of pancreatic cancer.

## METHODS

We performed a comprehensive literature search using the PubMed database. The keywords used were “genetic polymorphism” plus “pancreatic cancer.” In addition, we also cite the published literature addressing candidate gene polymorphisms and their associations with other cancer types, as well as findings from GWAS and experimental studies.

## RESULTS

### Tobacco smoking and genetic polymorphisms in carcinogen-metabolizing genes

Pancreatic cancer is a tobacco-induced cancer: epidemiologic studies have consistently shown that cigarette smoking increases the risk for pancreatic cancer. A meta-analysis of 82 case–control and cohort studies reported a 1.8-fold increased risk for current smokers as compared with nonsmokers.^5^ Tobacco smoke contains a variety of carcinogens, of which 4-(methylnitrosamino)-1-(3-pyridyl)-1-butanone and its metabolite 4-(methylnitrosamino)-1-(3-pyridyl)-1-butanol are the major carcinogens involved in pancreatic carcinogenesis.^15^ While the role of polycyclic aromatic hydrocarbons exposure and metabolism in pancreatic cancer needs further investigation, aromatic amine and heterocyclic amine have been implicated in the pathogenesis of pancreatic cancer.^16^

Metabolic activation of carcinogens forms DNA adducts, causing mutations in crucial genes, including *RAS*, *MYC*, *TP53*, and *P16*.^15^ The accumulation of these genetic mutations leads to uncontrolled cell growth and tumor development. Enzymes such as cytochrome P450S, glutathione-S-transferase (GST), N-acetyltransferase 1 (NAT1), and N-acetyltransferase (NAT2) are involved in the metabolic activation of carcinogens to DNA adducts and in detoxification to other products.^17^ Human cytochrome P450 1A1 (*CYP1A1*) encodes aryl hydrocarbon hydroxylase, a phase I enzyme involved in the activation of tobacco-related carcinogens.^17^ The GSTs are a family of phase II isoenzymes that are involved in phase II drug metabolism by conjugation of electrophilic substances with glutathione.^18^ GST detoxifies a broad range of substances, including carcinogens, environmental toxins, and drugs. Genetic polymorphisms resulting in lack of enzyme activity due to homozygous deletion of the *GSTM1* and *GSTT1* genes have been described.^18^ The frequencies of these deletions vary across populations.^19^ Molecular epidemiologic studies have shown increased risk for various cancers among individuals with the *NAT1* rapid acetylator or *NAT2* slow acetylator genotypes, in the presence of known carcinogen exposure, such as smoking or dietary exposure to heterocyclic amine.^20^

Although several molecular epidemiologic studies have examined the associations between variants of the genes encoding *CYP*, *GST*, and *NAT* enzymes and pancreatic cancer risk, most of the important findings regarding the main effects of genetic variations or gene–environment interactions were reported by 2 case–control studies: an ongoing hospital-based case–control study at MD Anderson Cancer Center^21^ and a population-based case–control study in 6 areas of San Francisco Bay from 1994 to 2001^8^ (Table 1). Both studies enrolled a relatively large number of case and control subjects, which allowed for an analysis of gene–environment interactions. The case–control study conducted at MD Anderson Cancer Center revealed the following. (1) *NAT1* rapid alleles were associated with 1.5-fold increased risk, and the effect was more prominent among ever smokers and females.^21^ A significant synergistic effect of the CYP1A2*1F allele and *NAT1* rapid alleles with respect to the risk for pancreatic cancer was also detected. (2) The rare NAT1*10 or NAT1*11-NAT2*6A diplotype may be an “at-risk” genetic variant for pancreatic cancer.^22^ (3) The GSTP1*C variant conferred a possible protective effect against pancreatic cancer.^23^ (4) A significant interaction was noted between *CYP1A2*, *NAT1*, and heavy smoking and dietary mutagen intake.^24^

**Table 1.** Summary of findings from case–control studies of genetic polymorphisms in carcinogen-metabolizing genes and their interactions with environmental factors and pancreatic cancer risk

Study and year	Study population	No. of cases	No. of controls	Genetic polymorphisms	Main effects of polymorphisms	Gene–environment interaction
Lee et al, 1997 (Ref 27)	Koreans	45	53	P-450 (1A1, 2D6, and 2E1)	No association	Unreported

Bartsch H, et al, 1998 (Ref 25)	Whites	81	78	NAT1, NAT2, GSTM1, NAD(P)H: NQO1	GSTM1 and NAT1 enzymes associated with modest increase in susceptibility to pancreatic cancer	Unreported

Liu et al, 2000 (Ref 26)	Canadian	149	146	CYP1A1, GSTM1, GSTT1	No association	Not observed

Duell EJ, et al, 2002 (Ref 8)	Whites	309	964	CYP1A1, GSTM1, GSTT1	No significant main effects	Never smokers with GSTT1-present genotype vs heavy smokers with GSTT1-null genotype: OR, 3.2 (95% CI, 1.3–8.1) for men and 5.0 (1.8–14.5) for women

Li et al (Ref 21)	Non-Hispanic whites	365	379	P4501A1, NAT	NAT1 rapid alleles associated with 1.5-fold increased risk	Interaction with smoking

Jiao et al, 2007 (Ref 22)	Non-Hispanic whites	352	315	GSTM1, GSTT1, GSTP1	No significant main effects	GSTP1*C variant conferred possible protective effect in older subjects

Jiao et al, 2007 (Ref 23)	Non-Hispanic whites	532	581	Haplotype of NAT1 and NAT2	Rare NAT1*10 or NAT*11-NAT2*6A diplotype associated with increased risk	Interactions between NAT2 slow genotype and smoking and history of diabetes

Suzuki et al, 2008 (Ref 24)	Non-Hispanic whites	755	636	P4501A2, SULT1A1, and NAT	No significant main effects	Interactions between CYP1A2, NAT1, and heavy smoking and dietary mutagen intake

Abbreviations: NAT, N-acetyltransferase; GSTM, glutathione-S-transferase; CYP1A1, cytochrome P450 1A1; OR, odds ratio; CI, confidence interval.

Results reported by Li et al, Jiao et al, and Suzuki et al came from the same research group.

In the population-based case–control study carried out in 6 areas of San Francisco Bay, Duell et al examined polymorphisms in carcinogen-metabolizing genes, smoking, and pancreatic cancer risk in whites and found that there was no significant increase in risk associated with any genotype examined.^8^ However, the odds ratio (OR) was 5.0 (95% confidence interval [CI], 1.8–14.5) for heavy smokers who had a deletion polymorphism in *GSTT1*, suggesting that inherited deletion polymorphisms in *GSTT1* increase susceptibility to smoking-related pancreatic cancer. Another notable finding from this study was that the interaction was stronger in women than in men. Although these results require replication in other studies, the findings suggest that women with a *GSTT1*-null or *GSTM1*-null genotype may be more susceptible than men to the effects of DNA adducts. In addition to the 2 studies mentioned above, other, small studies have addressed this issue, although the findings are difficult to interpret due to the small sample size.^25^^–^^27^

More studies are needed to examine genetic variations in tobacco-metabolizing genes and their associations with pancreatic cancer among different ethnic populations. However, based on current, limited evidence from molecular epidemiologic studies, it seems unlikely that individual polymorphisms themselves confer major susceptibility to pancreatic cancer. Given the reported synergistic effects of smoking and certain carcinogen-metabolizing gene polymorphisms, it is essential to clarify gene–environment interactions in future studies.

### DNA repair and polymorphisms in DNA repair genes

DNA repair plays a crucial role in cellular defense against mutations caused by carcinogens and endogenous mechanisms. Four types of DNA repair systems—nucleotide excision repair, base excision repair (BER), mismatch repair, and recombination repair—have been identified so far.^28^ Defects in these pathways may result in a predisposition to cancer. Each type of DNA repair is understood in considerable detail. We focus on BER because the genetic polymorphisms in this repair pathway are the most extensively studied in molecular epidemiologic studies. DNA bases are particularly susceptible to oxidation mediated by reactive oxygen species, which can be produced as a consequence of ionizing radiation or environmental exposure to transition metals, chemical oxidants, and free radicals. Reactive oxygen species have been linked to the initiation and progression of cancer.^29^ BER plays an important role in preventing mutations associated with a common product of oxidative damage to DNA, 8-oxoguanine. X-Ray Repair Cross-Complementing Group 1 (*XRCC1*), located on 19q13.2, is a polymorphic BER gene that has been the most extensively examined in molecular epidemiologic studies of the risk of various cancers.^30^ In the above-mentioned population-based case–control study conducted in 6 areas of San Francisco Bay,^31^ a synergistic effect between the *XRCC1 399*Gln allele and tobacco smoking in relation to pancreatic cancer risk was observed, although no significant associations were noted between *XRCC1* genotypes and pancreatic cancer risk. As compared with never-active smokers and passive smokers with the Arg/Arg genotype, the age- and race-adjusted ORs for heavy smokers (≥41 pack-years) with the Gln/Gln or Arg/Gln genotypes were 7.0 (95% CI, 2.4–21) in women and 2.4 (1.1–5.0) in men. The interaction suggests that *XRCC1* Arg399Gln and BER capacity are important in susceptibility to smoking-induced pancreatic cancer. However, these findings need to be confirmed in other studies, as the number of study subjects was small in the analysis exploring gene–environment interaction.

The 8-oxoguanine DNA glycosylase (*OGG1*) gene is another BER gene that removes oxidative DNA lesions.^28^ *OGG1* has been associated with altered risk of human cancers. In data from the hospital case–control study conducted at MD Anderson Center in the United States, Li et al noted significantly reduced overall survival in patients with the *OGG1* C315G (rs1052133) GG homozygous variant genotype.^32^ Furthermore, they reported a weak interaction of the *OGG1* C315G CC/CG genotype with diabetes in pancreatic cancer. These findings suggest that the CC/CG genotype, combined with environmental exposure, confers increased susceptibility to pancreatic cancer.

Li et al also examined associations of pancreatic cancer with selected DNA repair polymorphisms in other types of DNA repair pathways, including *XRCC2*, *XRCC3*,^33^ *RAD54L*, and *RecQ1* in the recombination repair pathway,^34^ and the xeroderma pigmentosum group D (*XPD*) in the NER pathway.^35^ They found that variant alleles of *XRCC2* R188H and *XRCC3* A17893G were associated with significantly reduced survival in pancreatic cancer patients and that *XRCC2* Arg188His polymorphisms may be genetic modifiers for smoking-related pancreatic cancer.

Overall, evidence from a small number of molecular epidemiologic studies supports a role for genetic variability in DNA repair in the risk for pancreatic cancer. Due to small sample sizes and heterogeneous study designs, however, the results are inconclusive and require confirmation. Because hundreds of genetic polymorphisms may be involved in maintaining genomic integrity, additional studies with large sample sizes are needed to elucidate multiple sequence variants in a gene or multiple genes within an entire pathway.

### Folate intake and polymorphisms in folate-metabolizing genes

Folate is a water-soluble B vitamin abundant in green leafy vegetables, citrus fruit, legumes, and cereals. Substantial evidence from epidemiologic and laboratory research supports a role for folate in carcinogenesis.^36^^,^^37^ Epidemiologic studies have consistently shown an inverse association between folate intake and pancreatic cancer risk. Based on a meta-analysis in which data from 4 cohort studies and 1 case–control study were analyzed, individuals with the highest folate intake had a 51% lower risk than those with the lowest folate intake.^38^ Mechanistic studies have elucidated 2 major underlying mechanisms that may be involved. Folate deficiency may induce misincorporation of uracil into DNA, leading to chromosomal breaks and mutations. In addition, folate deficiency may cause aberrant DNA methylation, resulting in altered expression of critical proto-oncogenes and tumor suppressor genes.^39^ Moreover, functional polymorphisms in folate-metabolizing genes may confer susceptibility to cancer. Among the several polymorphisms in the folate metabolic pathway, polymorphisms in the 5-10 methylenetetrahydrofolate reductase (*MTHFR*) gene are the most extensively studied. A central enzyme in folate metabolism, MTHFR irreversibly converts 5,10-methylenetetrahydrofolate to 5-methyltetrahydrofolate, the predominant form of folate in systemic circulation. Thus, *MTHFR* acts as a critical junction in folate metabolism by directing folate metabolites toward the DNA methylation pathway and away from the DNA synthesis pathway. Two common functional polymorphisms of the *MTHFR* gene, C677T and A1298C, have been identified.^39^ Regarding C677T, the TT genotype (variant type) has been shown to have 35% lower enzyme activity than the CC genotype (wild type).^39^ As for A1298C, homozygotes (CC) have approximately 60% of normal *MTHFR* activity.

Since 2005, five studies have reported an association between the *MTHFR* C677T genotype and pancreatic cancer risk, but the results were not consistent^9^^,^^40^^–^^43^ (Table 2). The TT genotype was associated with significantly increased risk for pancreatic cancer in 2 case–control studies carried out in the United States^9^ and China^40^: the ORs were 2.14 (95% CI, 1.14–4.01) and 5.12 (2.94–9.10), respectively. Notably, a significant interaction between TT genotype and smoking in pancreatic cancer risk was also observed. In these 2 studies, heavy smokers with the TT genotype had an approximately 7-fold increased risk as compared with nonsmokers with the CC genotype.^9^^,^^40^ In contrast, 2 Japanese case–control studies reported no increased risk associated with the TT genotype.^41^^,^^42^ In one of these studies, the OR for pancreatic cancer in individuals with the TT genotype was 0.75 (0.41–1.35), but the association was statistically insignificant.^42^ Due to the small number of studies and the wide heterogeneity in results, a summary OR could not be calculated in a meta-analysis of the *MTHFR* C677T genotype and pancreatic cancer risk, as only 3 published studies were included.^38^ Inadequate sample size and different criteria for control selection might have contributed to the inconsistent results reported so far. As for the A1298C genotype, evidence is insufficient to draw a conclusion: 2 studies showed no important effects on pancreatic cancer, and one study suggested a 1.8-fold increased risk in subjects with the CC genotype.^38^

**Table 2.** Summary of findings from case–control studies of genetic polymorphisms in folate-metabolizing genes and their interactions with environmental factors and pancreatic cancer risk

Study and year	Study population	No. of cases	No. of controls	Genetic polymorphisms	Main effects of polymorphisms^a^	Gene–environment interaction^a^
Li et al, 2005 (Ref 9)	Non-Hispanic US whites	347	348	*MTHFR* C677T, A1298C	Significant effect for C677T: CT, 0.90 (0.63–1.27), TT, 2.14 (1.14–4.01); no association for A1298C	Heavy smokers with TT vs never smokers with CC/CT: 6.83 (1.91–24.38) Heavy alcohol drinkers with TT vs nondrinkers with CC/CT: 4.23 (0.88–20.3)

Wang et al, 2005 (Ref 40)	Chinese	163	337	*MTHFR* C677T, A1298C, TS	Significant effect for C677T: CT, 2.60 (1.61–4.29), TT, 5.12 (2.94–9.10); no association for A1298C	Heavy smokers with TT vs never smokers with CC/CT: 6.69 (3.39–13.63) Alcohol drinkers with TT/CT vs nondrinkers with CC: 4.39 (2.25–8.78)

Matsubayashi et al, 2005 (Ref 41)	Americans	303	305	*MTHFR* C677T, A1298C	No association for C677T: CT, 0.79 (0.56–1.11), TT, 1.10 (0.67–1.82)	No significant interaction with smoking

Suzuki et al, 2008 (Ref 42)	Japanese	157	785	*MTHFR* C677T, MTR A2756G, TS variable number of tandem repeat	No association for C677T: CT, 0.98 (0.65–1.47), TT, 0.75 (0.41–1.35)	No significant interaction with alcohol drinking

Ohnami et al, 2008 (Ref 43)	Japanese	198	182	*MTHFRC677T*, MTRR (rs1801394, rs162049, rs10380)	No association for C677T, but MTRR polymorphisms associated with increased risk	No association

Abbreviations: MTHFR, methylenetetrahydrofolate reductase; MTR, methionine synthase; MTRR, methionine synthase reductase; OR, odds ratio.

^a^Values are odds ratios (95% confidence interval).

Given the strong interaction of the *MTHFR* genotype with environmental factors such as smoking and alcohol consumption, it is important to unravel their complex relationships in additional large, adequately powered studies. Furthermore, because the balance between the use of methylenetetrahydrofolate for DNA synthesis rather than for methionine synthesis might depend on the presence of the 677T variant of *MTHFR* and nutritional folate status, studies targeting populations with folate deficiency in developing countries may provide valuable information.

**Table 3.** Associations of genetic polymorphisms and pancreatic cancer risk that require assessment in future studies

Candidate genes	Selected polymorphisms	Potential interactions with environmental factors	Circulating biomarker
Vitamin D signaling	rs11574143	Sun exposure, diet	Plasma 25-hydroxyvitamin D
Melatonin receptors and clock genes	*MTNR1B*, rs10830963, rs11133373 in *CLOCK*	Diabetes	Plasma or urinary melatonin (6-sulfatoxymelatonin)
Insulin, IGF gene	IGF1 haplotype and the IGF2 Ex4 -233 C>T TT genotype	Diabetes, obesity	Plasma or serum IGF
TGF-β signaling	TGFBR1*6A	Diabetes	Plasma or serum TGF-β
Infection-related gene polymorphisms	COX-2 polymorphisms	N/A	N/A
ABO gene	rs505922	N/A	N/A
Genes in chromosome 13q22.1	Novel polymorphisms to be identified	N/A	N/A

Abbreviations: IGF, insulin-like growth factor; TGF-β, transforming growth factor-β; MTNR1B, melatonin receptor 1B; COX-2, cyclooxygenase-2.

N/A: not applicable.

### Alcohol consumption and polymorphisms in alcohol-metabolizing enzymes

Although the majority of prospective cohort studies found no significant increase in the risk of pancreatic cancer with moderate to high levels of alcohol intake in a general population,^7^ some evidence suggests that excessive drinking may increase risk in population subsets.^44^

Ethanol is mainly metabolized to acetaldehyde by alcohol dehydrogenase enzymes and further oxidized to acetate by acetaldehyde dehydrogenase. Acetaldehyde has been shown to have carcinogenic effects in experimental studies and is the main mechanism to explain alcohol-induced carcinogenesis.^45^ Variations in the production and/or oxidation of acetaldehyde among individuals are caused by single-nucleotide polymorphisms (SNPs) of *ADH1B*, *ADH1CI*, and *ALDH2*.^45^ In particular, people homozygous for *ALDH*2*2 display flush syndrome, which is characterized by nausea, vomiting, and facial flushing after ingestion of a small amount of alcohol.

Very few studies have addressed the role of polymorphisms in alcohol-metabolizing enzymes and pancreatic cancer risk. A recent case–control study involving 160 pancreatic cancer patients and 800 age- and sex-matched controls in Japan found that alcohol consumption was associated with increased risk in individuals with the *ALDH2* Lys+ allele or *ADH1B* His/His or *ADH1C* Arg/Arg genotypes, but not in those with the *ALDH2* Glu/Glu genotype or *ADH1B* Arg or *ADH1C* Gln alleles.^46^ This suggests that the risk of pancreatic cancer is associated with the combined effect of alcohol consumption and certain polymorphisms in alcohol-metabolizing enzymes.

Because the metabolism of alcohol and acetaldehyde is strongly influenced by alcohol-metabolizing enzymes, future molecular epidemiologic studies need to examine the effect of these polymorphisms on pancreatic cancer risk while accounting for alcohol consumption.

### Vitamin D and polymorphisms in vitamin D pathway genes

Humans get vitamin D mainly from exposure to sunlight or their diet. Vitamin D is metabolized in the liver to 25-hydroxyvitamin D [25(OH)D], which is further metabolized in the kidneys by the enzyme 25-hydroxivitamin D-1α-hydroxylase (CYP27B1) to its active form, 1,25-dihydroxyvitamin D.^47^ Vitamin D receptor, a crucial mediator of the cellular effects of vitamin D, is present in a variety of cell types, including pancreatic beta cells.^48^ Experimental evidence shows that vitamin D receptor interacts with other cell-signaling pathways to influence cancer development.^49^ Ecologic studies have linked sun exposure to lower pancreatic cancer mortality.^50^ Individuals with higher circulating 25(OH)D levels have been found to have decreased risks of breast, colorectal, and prostate cancer in numerous prospective studies.^51^ Given the collective evidence from epidemiologic and experimental studies, it is plausible that high vitamin D levels may be associated with a lower risk of pancreatic cancer. However, the role of vitamin D in the development of pancreatic cancer remains unclear due to the small number of studies.

High vitamin D exposure is hypothesized to decrease cancer risk, possibly through genomic effects modulated by the vitamin D receptor, and by autocrine/paracrine metabolism of the vitamin D receptor’s ligand, 1α,25-(OH)2-vitamin D3.^49^ Recently, an increasing number of studies have examined polymorphisms in vitamin D receptor and selected genes in the vitamin D pathway in relation to colorectal, breast, and prostate cancer risk.^48^ However, there is currently no strong, consistent epidemiologic evidence for a substantial influence of any single variant in vitamin D pathway genes on cancer risk. The association of pancreatic cancer with serum vitamin 25(OH)D levels and polymorphic variants in genes encoding for enzymes that synthesize, carry, and degrade vitamin D is an important research subject for future studies.

### Circadian disruption, melatonin, and genetic variations in clock genes

To date, no studies have examined the association between circadian disruption and pancreatic cancer risk in humans. Experimental data, however, have shown that disruption of circadian rhythms in mice is associated with accelerated growth of pancreatic cancer.^52^ Although the findings are not entirely consistent, epidemiologic studies have indicated that shift work is significantly associated with increased risks of breast, colorectal, and prostate cancer.^53^ On the basis of considerable evidence from animal studies and limited evidence from epidemiologic studies, the working group of IARC concluded in 2007 that “shift-work that involves circadian disruption is probably carcinogenic to humans.”^54^ The principal mechanism involves melatonin, a neurohormone that regulates the circadian rhythm.^55^ Three recent GWAS have shown that the common variant *MTNR1B* (melatonin receptor 1B) is associated with insulin and glucose concentrations.^56^^–^^58^ The melatonin receptor MT1 is highly expressed in pancreatic islet cells, and the expression of *MTNR1B* has been confirmed in both islets and sorted beta cells.^59^ Given the close relationship of hyperinsulinemia and diabetes with pancreatic cancer risk, it might prove interesting to examine the risk genotypes of *MTNR1B* and their interactions with diabetes and other environmental factors in pancreatic cancer development.

In addition to melatonin, circadian rhythms are controlled and maintained by several circadian genes via transcription–translation feedback loops that include positive activators, such as Clock, neuronal PAS domain protein 2 (*NPAS2*), cryptochrome 1 (*CRY1*) and *CRY2*, and period 1 (*PER1*), *PER2*, and *PER3*.^60^ To test the hypothesis that genetic variations in these genes may confer susceptibility to prostate cancer, Zhu et al genotyped a total of 41 tagging and amino acid-altering SNPs in 10 circadian genes in a population-based case–control study of white men and found that *NPAS2* showed the most robust association with prostate cancer risk.^61^ No studies, however, have examined genetic polymorphisms in clock genes and pancreatic cancer risk.

Because of the important role of melatonin and circadian genes in maintaining circadian rhythm, future studies may address genetic variations in these genes and the risk of pancreatic cancer.

### Insulin and insulin-like growth factor gene polymorphisms

Obesity and type II diabetes are well established risk factors for pancreatic cancer, especially in developed countries. Elevated levels of insulin and insulin-like growth factors (IGFs), such as IGF-I, are important mechanisms underlying the association between obesity, diabetes, and pancreatic cancer.^62^ Insulin, IGF-1, and the insulin receptor-related receptor can form functional hybrids.^63^ IGF1 and IGF1 receptors are highly expressed in pancreatic cancer cells, and IGF2 imprinting is disrupted in many tumors.^64^

Despite strong experimental evidence indicating that IGFs play an important role in carcinogenesis—including the regulation of cell proliferation, differentiation, and apoptosis—the results of epidemiologic studies examining IGFs in relation to cancer risk are less persuasive. Using data from a nested case–control study in the Japan Collaborative Cohort (JACC) Study, we found a positive association between baseline IGF-1 levels and the risk of pancreatic cancer mortality in apparently healthy Japanese.^65^ However, no significant associations were observed in other studies.^66^ Only 1 study has examined the association between genetic polymorphisms in IGF genes and pancreatic cancer risk.^67^ Of 6 SNPs of IGF1 and IGF2 that were examined in a case–control study by Suzuki et al, the IGF1 haplotype and the IGF2 Ex4 -233 C>T TT genotype were significantly associated with decreased risk of pancreatic cancer, which suggests that polymorphic variants of the IGF genes may serve as a susceptibility factor for pancreatic cancer. Future studies are warranted to explore polymorphisms in IGF gene pathways and their interaction with obesity and physical activity in pancreatic cancer risk.

### Transforming growth factor-β (TGF-β) and polymorphisms in the TGF-β pathway

TGF-β regulates tumor initiation, progression, and metastasis via its signaling pathway involving membrane receptors and SMAD transcription factors.^68^ The dual role of TGF-β in cancer, both as a tumor suppressor and tumor promoter, has been well defined.^69^ Several lines of evidence demonstrate that pancreatic cancer is clearly linked to TGF-β.^70^ In particular, SMAD4, a component of the TGF-β pathway, is mutated in approximately 50% of pancreatic cancers.^71^

Because of the presence of plausible mechanisms, a study of the associations of polymorphisms in TGF-β pathway with pancreatic cancer risk should prove interesting. TGFBR1*6A is emerging as a high frequency, low-penetrance tumor susceptibility allele that confers susceptibility to breast, ovarian, and colorectal cancer. A meta-analysis of 7 case–control studies of TGFBR1*6A and various cancer types combined showed that TGFBR1*6A carriers had a 26% increased risk.^72^ The role of TGFBR1*6A in pancreatic cancer remains unclear and is a subject of future study.

### Inflammation and infection-related gene polymorphisms

Inflammation has been implicated in pancreatic carcinogenesis. Cyclooxygenase-2 (COX2) is a key enzyme involved in biologic processes including inflammation, immune function, and cell proliferation.^73^ The overexpression of this enzyme has been shown in pancreatic cancer.^74^ However, there have been few studies addressing inflammation-related genetic polymorphisms and pancreatic cancer risk. Zhao et al showed that functional *COX-2* polymorphisms are associated with susceptibility to pancreatic cancer.^75^ In another case–control study, 3 infection-related polymorphisms (*TNF-A, RANTE5, and CCR5*) were examined, but no significant effects were found.^76^

*Helicobacter pylori* infection induces chronic inflammation and has been established as a risk factor for gastric cancer. It remains controversial, however, whether *H. pylori* infection plays a role in pancreatic cancer development.^77^ Epidemiologic studies examining this issue have produced mixed results. The hypothesis has been proposed that polymorphisms in genes involved in inflammatory response, such as *IL-1A*, *IL-1B*, *IL-6*, *IL-8*, may help explain why only a subset of individuals infected with *H. pylori* develops gastric cancer. Similarly, it is important to comprehensively analyze the effects of these polymorphisms on pancreatic cancer risk.

### SNPs indentified by genome-wide association studies

Genome-wide association studies (GWAS) have been proven to be a valuable tool for identifying common alleles that influence disease risk.^78^ Fast-evolving sequencing technology allows researchers to scan across the genome in a large set of cases and controls to identify new associations that link certain regions to disease risk.

The first GWAS for pancreatic cancer, published in *Nature Genetics* in August 2009, identified common risk variants that map to the first intron of the *ABO* gene on chromosome 9q34.2 (SNP rs505922).^11^ This finding implies that people with blood group O may have a lower risk than those with groups A or B.

Base on DNA collected from nearly 4000 patients in 13 different studies, a second GWAS for pancreatic cancer has, for the first time, identified pancreatic cancer susceptibility loci on 3 chromosomes—13q22.1, 1q32.1, and 5p15.33.^12^ Of the 3 regions, the locus on 13q22.1 appears to be specific for pancreatic cancer.

Findings from the 2 GWAS of pancreatic cancer have provided new insight into pancreatic cancer etiology; however, they need to be replicated in other large studies. Furthermore, follow-up studies after the GWAS must address whether SNPs discovered by GWAS represent functional variants or simply tag true variants located in the same haplotype.

### Mitochondrial genetic polymorphisms

Mitochondria play an important role in cellular energy metabolism, free radical generation, and apoptosis. Mitochondrial DNA mutations can initiate a cascade of events leading to persistent oxidative stress, a condition that probably favors tumor development.^80^ Although previous studies have examined the association between mitochondrial genetic polymorphisms and pancreatic cancer,^81^^–^^83^ the results were inconsistent. While a mitochondrial SNP in the 16519 mitochondrial DNA nucleotide was found to be associated with worse prognosis,^81^ this positive association was not replicated in a recent study involving 990 pancreatic cancer patients.^82^ A recent large case–control study comprised 955 participants with primary pancreatic adenocarcinoma and 1102 control subjects and examined 24 mitochondrial SNPs and 11 common haplogroups, none of which was significantly associated with pancreatic cancer risk.^83^ Their results did not support the significant involvement of mitochondrial SNPs or haplogroups in the development of pancreatic cancer. Because of the important role of mitochondrial DNA in cancer, further investigations of mitochondrial genetic variations are necessary to provide insight into the etiology of pancreatic cancer.

## DISCUSSION

Molecular epidemiologic studies examining the associations between polymorphisms in several gene pathways and pancreatic cancer risk have produced mixed results. Overall, individual polymorphisms did not seem to confer marked susceptibility; however, some studies implicated interactions of polymorphisms in carcinogen-metabolizing genes, DNA repair genes, and folate-metabolizing genes with smoking, diet, and obesity. The principal weakness of these studies is small sample size; thus, it is difficult to detect statistically significant gene–gene or gene–environment interactions. Because of this, no functional variants reported so far have been used to predict pancreatic cancer risk in the clinical setting. Another critical challenge is that the measurement of environmental influence in epidemiologic studies must be improved to better define gene–environment interaction.

Two recent GWAS of pancreatic cancer have provided intriguing results that need to be confirmed in additional studies. With the decreasing cost of genotype sequencing, we expect that future GWAS will unravel causal variants with significant effects on pancreatic cancer. Hopefully, disease susceptibility variants will be discovered from GWAS, and the interactions of these variants with environmental factors will be more frequently confirmed in molecular epidemiologic studies. Since we have yet to discover rare variants that greatly increase the risk of pancreatic cancer, perhaps, as is the case with other complex diseases, common low-risk variants in different genes act collectively to confer susceptibility to pancreatic cancer in individuals who have repeated environmental exposures, such as smoking and intake of red meat. A recent study provided critical evidence to support this notion by demonstrating that pancreatic cancer results from genetic alterations of a large number of genes that function through 12 pathways and processes,^79^ including TGF-β signaling and DNA damage control, which were discussed in this review.

What is the future direction for research on the etiology of pancreatic cancer? First, we believe that unraveling the functional SNP variants in a number of identified gene pathways, combined with novel variants identified in GWAS, is essential in deepening our understanding of pancreatic cancer risk. To achieve this goal, the complex gene–gene and gene–environmental interactions must be clarified in a rigorously designed molecular epidemiologic study with a large sample size. Second, in addition to SNPs, there is increasing recognition of the role of genetic variations—such as DNA copy number variations and variable-number tandem repeats—in cancer predisposition.^84^ High-resolution SNP arrays have made it possible to identify copy number variations. Moreover, there have been studies linking copy number variations and variable-number tandem repeats to pancreatic cancer risk.^85^^,^^86^ Elucidating these associations is an important goal for future research.
